# Maxi‐K channel (BK_C__a_) activity veils the myogenic tone of mesenteric artery in rats

**DOI:** 10.14814/phy2.13330

**Published:** 2017-07-18

**Authors:** Eun Yeong Suh, Ming Zhe Yin, Haiyue Lin, Yin Hua Zhang, Hae Young Yoo, Sung Joon Kim

**Affiliations:** ^1^ Departments of Physiology and of Biomedical Sciences Seoul National University College of Medicine Seoul Korea; ^2^ Chung‐Ang University Red Cross College of Nursing Seoul Korea

**Keywords:** BK_Ca_, K^+^ channel, mesenteric artery, myogenic tone, vascular smooth muscle cell

## Abstract

Arterioles and small arteries change their tone in response to transmural pressure changes, called myogenic tone (MT). In comparison to the branches of cerebral arteries (CA) showing prominent MT, the third branches of mesenteric arteries (MA) with similar diameters show weaker MT. Here, we aimed to analyze the electrophysiological differences responsible for the weaker MT in MA (MT_MA_) than MT in CA (MT_CA_). We measured ionic current using patch clamp in isolated MA smooth muscle cells (MASMCs) and CA smooth muscle cells (CASMCs) of rats. MT was analyzed using video analysis of pressurized small arteries. Quantitative‐PCR (q‐PCR) and immunofluorescence confocal microscopy were used to compare the mRNA and protein expression level of big‐conductance Ca^2+^‐activated K^+^ channel (BK_C__a_) subunits (Slo1*α* and Slo*β*1). Whole‐cell patch clamp study revealed higher density of voltage‐operated Ca^2+^ channel current (I_C__aV_) in the MASMCs than in CASMCs. Although voltage‐gated K^+^ channel current (I_K__v_) was also higher in MASMCs, treatment with Kv inhibitor (4‐aminopyridine) did not affect MT_MA_. Interestingly, BK_C__a_ current density and the frequency of spontaneous transient outward currents (STOCs) were consistently higher in MASMCs than in CASMCs. Inside‐out patch clamp showed that the Ca^2+^‐sensitivity of BK_C__a_ is higher in MASMCs than in CASMCs. Iberiotoxin, a selective BK_C__a_ inhibitor, augmented MT_MA_ by a larger extent than MT_CA_. Although q‐PCR analysis did not reveal a significant difference of mRNAs for Slo1*α* and Slo*β*1, immunofluorescence image suggested higher expression of Slo1*α* in MASMCs than in CASMCs. Despite the large I_C__aV_ density, the high activities of BK_C__a_ including the more frequent STOCs in MASMCs veils the potentially strong MT_MA_.

## Introduction

Small arteries and arterioles show contractile responses to an increase in luminal pressure (*P*_lum_). Since the contraction occurs independent of neurohumoral influences, it is named as myogenic tone (MT) of arteries. MT contributes to the peripheral resistance of vascular system, and thus blood pressure (Hill et al. 2006). Also, MT enables fine regulation of arterial diameter for constant regional blood flow despite the fluctuation of perfusion pressure, called autoregulation (Johnson 1977). The autoregulation of blood flow is observed in various organs such as brain and kidney. Among various types of vessels, cerebral arteries (CA) has been intensely investigated with regard to the mechanisms and pathophysiological implication of MT, reflecting physiological importance of autoregulation in the cerebral blood flow (Cipolla et al. 2014). Skeletal arteries such as cremaster artery and deep femoral artery also show robust MT (Kotecha and Hill 2005; Baek et al. 2010).

In the intestinal circulation, mesenteric arterial branches pierce the muscle layer to form microcirculatory networks in submucosa. It was reported that major part of the pressure drop in the intestine occurs in the precapillary arterioles. However, a significant pressure drop in the mesenteric arteries (MA) has indicated that MAs are not simple conduit but have active control of resistance (Lundgren 1989). In fact, previous studies have shown that the small branches of MA show MT (Sun et al. 1992; Enouri et al. 2011). Nevertheless, a closer inspection of literature reveals that the MT of MA (MT_MA_) is less consistent in the absence of agonists (VanBavel et al. 1991; Chlopicki et al. 2001; Mathewson and Dunn 2014). Also, MT_MA_ appears to be lower than the MT of CA (MT_CA_) and of skeletal arteries (Lagaud et al. 1999, 2002; Baek et al. 2010).

The relatively low MT_MA_ might be due to weak mechanosensation or effector functions in the mesenteric arterial smooth muscle cells (MASMCs). Since the wall tension changes induced by the increased luminal pressure (*P*_lum_) is accompanied by membrane depolarization and activation of voltage‐gated Ca^2+^ channels (CaV) in the smooth muscle cells (Harder 1984; Wesselman et al. 1996), differential activities of CaV might be responsible for the different levels of MT. Another plausible difference might be the activities of K^+^ channels; hyperpolarizing influence from K^+^ channels would attenuate MT via antagonizing inward cationic conductances including CaV. Voltage‐gated K^+^ channels (Kv) and big‐conductance Ca^2+^‐activated K^+^ channel (BK_Ca_) are commonly expressed in the most types of arterial myocytes (Standen and Quayle 1998). In addition, inwardly rectifying K^+^ channels (Kir) also play important roles in the fine regulation of resistance arteries including CA. However, the expression of Kir is not significant in MASMCs (Smith et al. 2008; Jin et al. 2011; Sonkusare et al. 2016).

The density of BK_Ca_ current and the expression of corresponding proteins (Slo1) are generally higher in smooth muscle cells than other cell types (Hu and Zhang 2012). BK_Ca_ are concertedly activated by increased cytosolic Ca^2+^ concentration ([Ca^2+^]_c_) and membrane depolarization. BK_Ca_ activity not only reflects the overall [Ca^2+^]_c_, but also the dynamic changes of subsarcolemmal concentration [Ca^2+^]_sl_. From the early period of patch clamp studies in both visceral and vascular smooth muscle cells, spontaneous transient outward currents (STOCs) lasting about 100 msec have been observed under physiological intracellular Ca^2+^ buffering conditions. The STOCs are thought to represent the simultaneous opening of up to a hundred BK_Ca_ (Benham and Bolton 1986) . Combined with [Ca^2+^]_c_ measurement studies, STOCs are generally accepted to reflect localized events of strong increase in the [Ca^2+^]_sl_, called Ca^2+^ sparks (Bolton and Imaizumi 1996). The hyperpolarizing influence of averaged STOCs contributes to the level of the arterial tone via setting the membrane potential (Pérez et al. 1999). Thus, comparative analysis of BK_Ca_ and their roles between different arteries are worth investigating to understand the functional differences between vessels (Hill et al. 2010). A previous study showed higher Ca^2+^ sensitivity of BK_Ca_ and higher expression of Slo‐1 beta subunit in cerebral arterial myocytes (CASMCs) than skeletal arterial smooth muscle cells (SkASMCs) (Yang et al. 2013). Even between the CA branches, the relatively large pial arteries show more influential role of BK_Ca_ than the smaller parenchymal arteries (Cipolla et al. 2014). However, the comparative study of BK_Ca_ in MASMCs with other arterial myocytes is lacking, especially related with the differential levels of MT.

On these backgrounds, here we investigate the activities of CaV, Kv, and BK_Ca_ currents in MASMCs and CASMCs. Along with the electrophysiological data, effective augmentation of smaller MT_MA_ by BK_Ca_ inhibitor suggest that higher activities of BK_Ca_ in MASMCs than CASMCs exert prominent auto‐inhibitory effect on MA.

## Materials and Methods

### Experimental animals

Male Sprague–Dawley (S–D) rats (8 weeks old) were fully anesthetized with pentobarbital sodium (100 mg/kg ip) and immediately sacrificed by decapitation. And their brain and gastrointestinal tract with mesentery arcade were moved into a normal Tyrode's (NT) solution. The study protocol was in accordance with the Guide for the Care and Use of Laboratory Animals published by the US National Institutes of Health (NIH Publication Eighth edition, revised 2011), and also conforms to the Institutional Animal Care and Use Committee (IACUC) in Seoul National University (IACUC approval No.: SNU‐111214‐4‐1)

### Measurement of vascular myogenic tone in resistance arteries

The third and fourth‐order branches of mesenteric arteries and the first‐branches of posterior and cerebellar arteries (200–250 *μ*m) were carefully dissected under the stereomicroscope. The arterial segments were placed in a glass‐bottomed vessel chamber (Model CH/1/SH; Living Systems Instrumentation, Burlington, VT). The vessel chamber contained NT and its composition was as follows (in mmol/L): 141.4 NaCl, 4 KCl, 1 MgCl_2_, 1.8 CaCl_2_, 0.33 NaH_2_PO_4_, 10 Glucose, and 10 HEPES (4‐(2‐hydroxyethyl)‐1‐piperazineethanesulfonic acid), adjusted to pH 7.4 with NaOH. The temperature was set at 37–38°C using a controller (Model TC‐01; Living Systems Instrumentation). The vessels were cannulated using glass micropipettes and secured with 12–0 nylon suture. Inner diameters (D_in_) of pressurized vessels were measured using a UBS CCD camera (DMK 41AU02, The Imaging Source, Bremen, Germany) and DMTVAS 6.2 software (IonOptix LLC, Milton, MA, USA). The value of luminal pressure (*P*_lum_) was set to 60 mmHg for at least an additional 40 min; spontaneous tone (i.e., decrease in vessel diameter) developed within this time frame and reached a steady state. Arterial segments without stable tone were discarded. After confirming the generation of myogenic tone, *P*_lum_ was lowered to 30 mmHg and incubated for equilibrium, the increase to 60 and 100 mmHg evaluate the MT. On the step‐like increase in *P*_lum_, *D*_in_ initially increased and then slowly decreased. To quantify MT of arteries, the maximal passive diameter (*D*_max,0Ca_) was measured in Ca^2+^‐free NT solution (0Ca‐NT) with 1 mmol/L EGTA (ethylene glycol‐bis(2‐aminoethylether)‐N,N,N′,N′‐tetraacetic acid). The maximal increase in *D*_in_ (Δ*D*_max_) by changing to the 0Ca‐NT was divided by *D*_max,0Ca_ (Δ*D*_max_/*D*_max,0Ca_), resulting the MT (%) at each *P*_lum_.

### Vascular smooth muscle cell isolation

After the dissection of arteries, vessels were initially incubated in 1 mL of the digestion medium I (0Ca‐NT solution containing 1 mg/mL papain) for 10–15 min and changed to the digestion medium II (0Ca‐NT solution containing 3 mg/mL collagenase) for another 10–15 min. Both digestion media contained bovine serum albumin (1 mg/mL) and dithiothreitol (1 mg/mL). The enzyme‐treated arteries were then gently agitated using a fire‐polished glass pipette and in a K^+^‐rich solution modified from KB solution (Isenberg and Klockner 1982), that contained (in mmol/L): 70 KOH, 50 l‐glutamate, 55 KCl, 20 taurine, 20 KH_2_PO_4_, 3 MgCl_2_, 20 Glucose, 10 HEPES, and 0.5 EGTA, adjusted to pH 7.3 with KOH.

### Electrophysiological recording

Isolated cells were transferred to a small chamber (0.2 mL) on the stage of an inverted microscope (IX‐70; Olympus, Osaka, Japan). The conventional whole‐cell or inside‐out configuration was performed with a patch‐clamp amplifier (Axopatch‐1C; Axon Instruments, Foster City, CA) and voltage‐clamp experiments were performed at room temperature (22–25°C). Membrane currents were recorded using the glass microelectrode with a resistance of 2–2.5 MΩ and 7–8 MΩ for whole‐cell and inside‐out patch clamp recordings, respectively. The signals were filtered at 5.0 kHz and sampled at a rate of 2.0 kHz and 10.0 kHz for whole‐cell and inside‐out patch clamp recordings, respectively. The pCLAMP software v.9.2 and Digidata‐1440A (Axon Instruments) were used to acquire data and apply command pulses.

The name and composition of the experimental solutions used in the electrophysiological recording are as below. The bath solution for recording CaV (whole‐cell) current contained the following (in mmol/L): 120 NaCl, 5 CsCl, 10 HEPES, 10 Glucose, 10 BaCl_2_, 10 tetraethylammonium chloride (TEA‐Cl), 0.5 MgCl_2_, adjusted to pH 7.4 with NaOH. The pipette solution for recording the CaV current contained (in mmol/L): 20 CsCl, 10 HEPES, 1 MgCl_2_, 5 EGTA, 120 Aspartic acid (Asp), 3 Mg‐ATP, adjusted to pH 7.2 with CsOH. To record the Kv current (whole‐cell), the NT solution contained 1 mmol/L TEA was used as bath solution. The pipette solution for recording the Kv contained the following (in mmol/L): 20 KCl, 10 HEPES, 1 MgCl_2_, 5 EGTA, 120 Asp, 3 Mg‐ATP, adjusted to pH 7.2 with KOH. To investigate the BK_Ca_ current (whole‐cell), we used the NT as bath solution. The pipette solution contained (in mmol/L): 20 KCl, 10 HEPES, 1 MgCl_2_, 120 Asp, 3 Mg‐ATP; pH was adjusted to 7.2 with KOH. And free Ca^2+^ activity in the pipette solution was clamped to 1 *μ*mol/L using 10 mmol/L EGTA and CaCl_2_ with WinMAX program (Chris Patton, Stanford University, http://www.stanford.edu/~cpatton/maxc.html). For recording STOCs (whole‐cell), we used the NT as bath solution. The pipette solution contained (in mmol/L): 20 KCl, 10 HEPES, 1 MgCl_2_, 0.1 EGTA, 120 Asp, 3 Mg‐ATP; pH was adjusted to 7.2 with KOH. To analyze the single‐channel conductance of BK_Ca_ (inside‐out), the bath solution contained the following (in mmol/L): 140 KCl, 10 HEPES, 10 Glucose, 1 MgCl_2_; pH was adjusted to 7.2 with KOH. And we added 10 mmol/L EGTA and CaCl_2_ in the bath solution to fix at 1 *μ*mol/L free Ca^2+^. The pipette solution contained the following (in mmol/L): 140 KCl, 10 HEPES, 10 Glucose, 1 MgCl_2_; pH was adjusted to 7.4 with KOH. To investigate Ca^2+^‐dependent activation of BK_Ca_ (inside‐out), the bath solution contained (in mmol/L): 140 KCl, 10 HEPES, 10 Glucose, 1 MgCl_2_, adjusted pH to 7.2 with KOH; variable amounts of CaCl_2_ were added to the bath solution to obtain 3, 10, and 30 *μ*mol/L free Ca^2+^. We made 3 and 10 *μ*mol/L free Ca^2+^ solution using 10 mmol/L of N‐(2‐Hydroxyethyl) ethylenediamine‐N,N′,N′‐triacetic acid (HEDTA) and CaCl_2_ calculated with the WinMAX program. For making 30 *μ*mol/L free Ca^2+^ solution, we simply added CaCl_2_ to the bath solution. We used NT as pipette solution.

### Real‐time quantitative PCR

Total RNA was extracted using TRizol reagent (Invitrogen), and its purified concentration was determined NanoDrop at 260 nm. The extracted RNA was reverse transcripted into cDNA using the PrimeScript RT Master Mix Perfect Real Time (Takara) according to the manufacturer's instructions. The cDNA was amplified using the primers as follows: GAPDH (forward, 5′‐ACGGCAAATTCAACGGCACAGTCA; reverse, 5′‐TGGGGGCATCGGCAGAAGG; BK_Ca_ *α*‐subunit (Slo1*α*, forward, 5′‐AAACAAGTAATTCCATCAAGCTGGTG; reverse, 5′‐CGTAAGTGCCTGGTTGTTTTGG); BK_Ca_ *β*1‐subunit (Slo*β*1, forward, 5′‐GTCTGCATCTTTGGGGATGT; reverse, 5′‐GGGGAAGGTGTGCAGTGTTT). cDNAs were amplified with SYBR Green (TOPreal qPCR 23 PreMIX) using a real‐time PCR system (Applied Biosystems 7500 real‐time PCR) under the conditions 95°C for 10 min, 40 cycles at 95°C for 10 sec, 55°C for 15 sec, and 72°C for 20 sec. After the final PCR cycle, a melting curve analysis was performed to analyze the specificity of the reaction. Relative gene expression was calculated using the comparative threshold (Ct) method (2^−∆∆Ct^).

### Immunofluorescence confocal microscopy

Fresh isolated MASCMs and CASMCs were fixed with 4% paraformaldehyde for 30 min and permeabilized with 0.1% Triton X‐100 for 15 min at room temperature. Then cells were blocked with 10% FBS for 1 h at room temperature, and incubated with primary antibodies of anti‐Slo1*α*‐subunit (1:50 diluted in 5% FBS, Alomone labs) or anti‐Slo*β*1‐subunit (1:200 diluted in 5% FBS, Abcam) overnight at 4°C. The channels expression levels were detected using Alexa Fluor 488‐conjugated goat anti‐rabbit secondary antibody (1:500 diluted with 5% FBS, Invitrogen). Imaged were acquired with Olympus FV1000 confocal microscope with a ×100 oil immersion objective.

### Drugs and chemicals

All drugs and chemicals used in this study were purchased from Sigma Chemical Co. (St Louis, MO) excluding iberiotoxin. Iberiotoxin was purchased from Tocris (Ellisville, MO).

### Statistical analysis

Data are shown as means ± SEM with the number of tested arteries indicated as *n*. Student's unpaired t‐test was used to test for significance at the level of 0.05.

## Results

**Figure 1:**
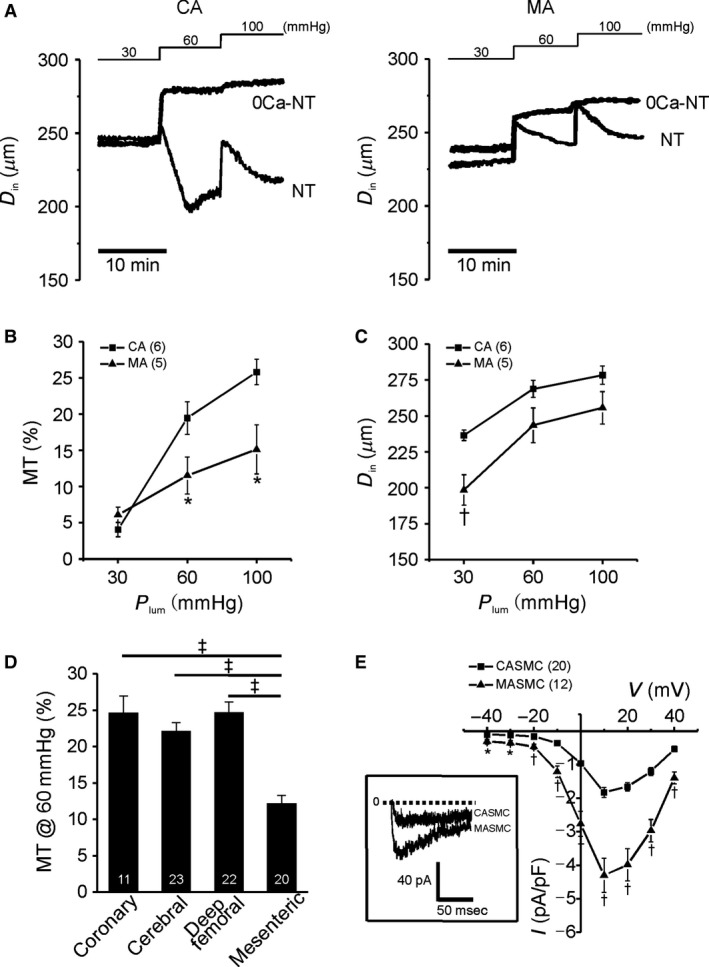
Low myogenic tone in MA despite relatively high *I*_C__aV_ in MASMCs. (A) Representative traces of *D*_in_ change in response to stepwise increase in *P*_lum_ (left; CA, right; MA). (B) Myogenic tone normalized to maximal passive *D*_in_ (*D*_max,0Ca_) from CA and MA (*P < 0.05). (C) Summary of the *D*_max,0Ca_ recorded in Ca^2+^ free condition (0Ca‐NT) (^†^ *P* < 0.01). (D) Comparison of myogenic tone at 60 mmHg in different type of arteries (coronary, cerebral, deep femoral, mesenteric) (^‡^ *P* < 0.001). (E) Summary of nifedipine‐sensitive *I*_C__aV_ in CASMCs and MASMCs (*P < 0.05). Representative recording at depolarizing membrane voltage (+10 mV) obtained by Cs^+^‐rich pipette and Ba^2+^ containing NT bath solution was shown in the inset (left panel of E) (*P < 0.05).

Representative traces of CA and D_in_ during the step‐like increase *P*_lum_ from 30 to 60 and 100 mmHg showed rebound contractions following initial passive dilation, consistent with the presence of MT (Fig. 1A). In the 0Ca‐NT, only the passive dilation was commonly observed on the *P*_lum_ increase (Fig. 1A). The MT_MA_ was significantly smaller than MT_CA_ (Fig. 1B). It was notable that the passive diameters of MA (3rd and 4th branches) used in this study were generally smaller than those of CA, especially at 30 mmHg of *P*_lum_ (Fig. 1C, Ca^2+^‐free condition). We also compared MT_MA_ with the MT of coronary and deep femoral arteries; MT_MA_ was consistently lower than the MT from the other types of arteries (Fig. 1D).

I_CaV_ was analyzed in MASMCs and CASMCs using the whole‐cell patch‐clamp technique using CsCl pipette solution. Step‐like depolarizations were applied from −70 mV of holding voltage, which revealed voltage‐dependent activation of inward currents (Fig. 1E, inset). Peak amplitudes of *I*_CaV_ versus recording voltages were plotted (*I*/*V* curve). Throughout the tested voltages, the density of *I*_CaV_ (pA/pF) was larger in MASMCs than CASMCs (Fig. 1E).

**Figure 2:**
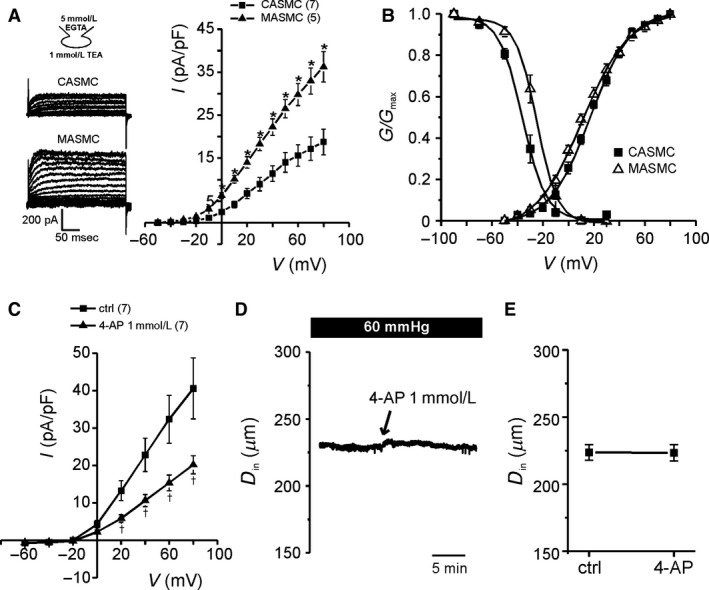
Higher amplitudes of I_K__v_ in MASMCs than CASMCs. (A) Left: representative Kv current recorded in CASMCs and MASMCs at voltage step from −50 mV to + 80 mV (10 mV‐increment, 300‐msec duration). To exclude the BK_C__a_ current, TEA 1 mmol/L was added in bath solution. Right: summary of normalized amplitude of TEA‐sensitive Kv current from CASMCs and MASMCs (*P < 0.05). (B) Conductance‐voltage relations of steady‐state activation and inactivation. The smooth curves were fitted to Boltzmann equation. Half activation voltage (*V*_1/2_) and slope value (*k*) were 11 ± 2.2 and 18 ± 0.9 mV for MASMCs (open triangle symbol) and 17 ± 1.7 and 16 ± 0.6 mV for CASMCs (filled square symbol). The *V*_1/2_ and *k* of the steady‐state inactivation were −25 ± 3.0 and 7.3 ± 0.4 mV for MASMCs and −35 ± 2.5 and 7.6 ± 0.3 mV for CASMCs, respectively. (C) Inhibition of *I*_K__v_ by 1 mmol/L 4‐AP in MASMCs (^†^ *P* < 0.01). (D) An original trace of *D*_in_ recording from pressurized MA in response to 1 mmol/L 4‐AP. (E) Summary of recorded *D*_in_ values under 1 mmol/L 4‐AP treatment in MA.

Next we analyzed the K^+^ channel currents, *I*_Kv_ and *I*_BKCa_, using KCl‐rich pipette solution. To selectively record *I*_Kv_, 5 mmol/L EGTA was included in the pipette solution and 1 mmol/L TEA was added to the bath solution. From −90 mV of holding voltage, step‐like depolarizing pulses were applied, which induced outward current from above −40 mV. The density of peak *I*_Kv_ (pA/pF) was about twofold higher in MASMCs than CASMCs (Fig. 2A). Voltage‐dependent steady‐state activation and inactivation of *I*_Kv_ were also analyzed. For the activation kinetics, peak Kv currents at test voltages were converted to conductance (g) and normalized to the maximum conductance at +80 mV (g/g_max_). The conductance to voltage relations were fitted to Boltzmann equation to obtain the half‐activation voltages, that were not different between MASMCs and CASMCs (Fig. 2B). For the inactivation kinetics, double‐pulse protocol was applied. The peak currents were measured at a 100 msec test voltage of +40 mV after 15 sec preconditioning voltages from −90 to +30 mV. The steady‐state inactivation curve was fitted with the Boltzmann equation. The fitted parameters are provided in Figure 2 legend. The analyses revealed that the voltage‐dependent inactivation of *I*_Kv_ occurs at more depolarized ranges in MASMCs than in CASMCs (Fig. 2B). The inactivation kinetics of *I*_Kv_ suggested that the steady‐state conductance of Kv in MASMCs would be higher than CASMCs at the depolarized membrane voltages above the threshold of Kv activation (Fig. 2B).

To investigate the contribution of Kv activity to MT_MA_, a Kv inhibitor, 4‐aminopyridine (4‐AP) was applied. The amplitudes of *I*_Kv_ was decreased to about 50% of control by 1 mmol/L of 4‐AP, respectively (Fig. 2C). However, the D_in_ of pressurized MA at 60 mmHg was not significantly affected by the treatment with 1 mmol/L 4‐AP (Fig. 2D and E).

**Figure 3:**
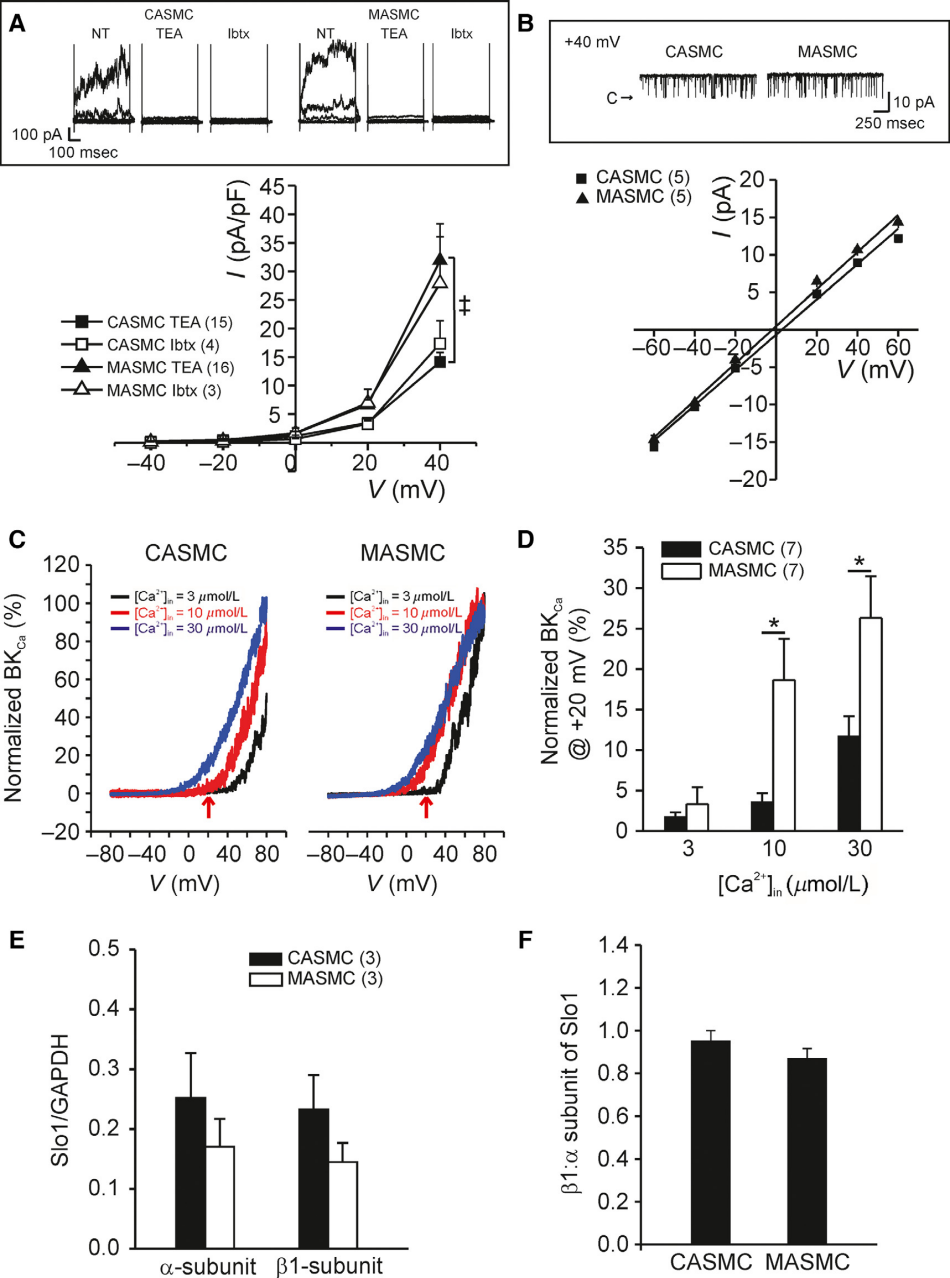
Electrophysiological property of BK_C__a_ channel and qPCR analysis of Slo1*α*, Slo*β*1‐subunit from isolated CASMCs and MASMCs. (A) Upper: representative current traces in whole‐cell configuration with fixed intracellular Ca^2+^ activity to 1 *μ*mol/L. Outward currents were mostly inhibited by 1 mmol/L TEA and 100 nmol/L iberiotoxin. Lower: summary of TEA‐sensitive and iberiotoxin (Ibtx)‐sensitive BK_C__a_ current density in CASMCs and MASMCs (^‡^ *P* < 0.001). (B) Upper: representative unitary current recorded at +40 mV in inside‐out configuration with intracellular free Ca^2+^ of 1 *μ*mol/L. Lower: unitary current–voltage (I–V) relationship of BK_C__a_ channels in CASMCs and MASMCs. (C) Normalized current–voltage relationship with maximum current at +80 mV in free Ca^2+^ 30 *μ*mol/L concentration. (D) Summary of the normalized BK_C__a_ current at +20 mV in response to a three range of free Ca^2+^ concentrations (3, 10 and 30 *μ*mol/L). (E) Bar graph showing the relative mRNA abundance of Slo1*α* and Slo*β*1 analyzed with 2^−∆∆Ct^ between CASMCs and MASMCs. (F) Ratio of Slo*β*1: Slo1*α* BK_C__a_ subunit mRNA expression between CASMCs and MASMCs.

Then we analyzed the BK_Ca_ activity to compare between MASMCs and CASMCs. With 1 *μ*mol/L intracellular free Ca^2+^ activity buffered by 10 mmol/L EGTA, the membrane voltage was held at −10 mV to induce the inactivation of Kv. Step‐like voltage changes were applied up to +40 mV, which induced outward currents with fluctuating noise. Most of the outward currents were abolished by 1 mmol/L TEA and 100 nmol/L iberiotoxin, relatively selective blockers of BK_Ca_ at this concentration (Fig. 3A, upper panel). The TEA‐sensitive and iberiotoxin‐sensitive BK_Ca_ currents were significantly higher in MASMCs than in CASMCs (Fig. 3A, lower panel).

Single channel activities of BK_Ca_ were analyzed under the inside‐out patch‐clamp conditions with 1 *μ*mol/L free Ca^2+^ activity at the cytoplasmic side (Fig. 3B, upper panel). Typical large amplitudes of unitary current with slope conductances around 240 pS were commonly observed in MASMCs and CASMCs (MASMC; 247 ± 7.5 pS, CASMC; 236 ± 7.4 pS, Fig. 3B). The Ca^2+^‐dependent activation of BK_Ca_ resides on the sensitization of their voltage‐dependent gating property with raised [Ca^2+^]_c_ (Cui et al. 2009; Carvalho‐de‐Souza et al. 2013). Therefore we tested whether the voltage‐dependent activation of BK_Ca_ at fixed [Ca^2+^]_c_ is different between MASMCs and CASMCs. In the inside‐out patches with multiple BK_Ca_, ramp‐like depolarizations from −80 to +80 mV were applied with 3, 10 and 30 *μ*mol/L [Ca^2+^]_c_ (Fig. 3C). Since the number of BK_Ca_ in the individual membrane patch is variable, the current–voltage relation was normalized to the maximum value at +80 mV with 30 *μ*mol/L [Ca^2+^]_c_. The normalized values commonly indicated that the Ca^2+^‐dependent voltage sensitivity of BK_Ca_ is higher in MASMCs than in CASMCs (Fig 3C and D).

**Figure 4:**
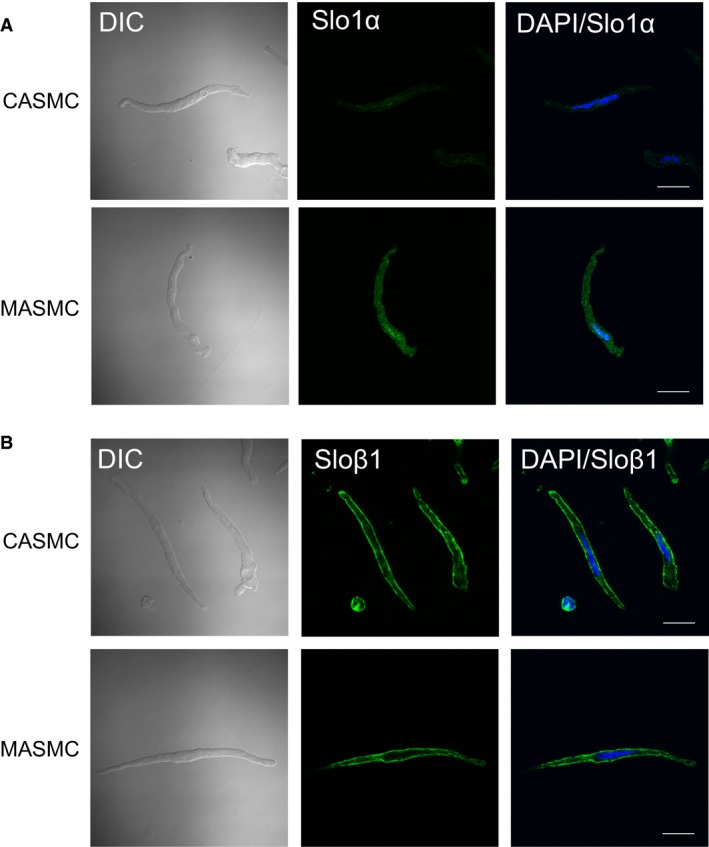
Representative immunofluorescence images of Slo1*α*‐ and Slo*β*1‐subunits in CASMCs and MASMCs. (A–B) Left: differential interference contrast (DIC) image of cells. Middle: staining of Slo1*α*‐ or Slo*β*1‐subunit in green. Right: Overlay with DAPI staining for nucleus (blue). Origianl magnification (×100). Scale bars, 20 *μ*m.

Despite the significant difference in the current density and voltage‐dependency of BK_Ca_, *α* and *β*1‐subunits of BK_Ca_ mRNA levels showed no difference between isolated CASMCs and MASMCs (Fig. 3E and F). To get a clue whether the protein expression of BK_Ca_, *α* and *β*1‐subunits is different between MAMSCs and CASMCs, we performed immunofluorescence confocal microcopy. According to the microscopic images, the BK_Ca_ *α*‐subunit specific fluorescence signals (Fig. 4A) in MASMCs seemed to higher than in CASMCs. In contrast, the *β*1‐subunit showed no significant difference (Fig. 4B).

**Figure 5:**
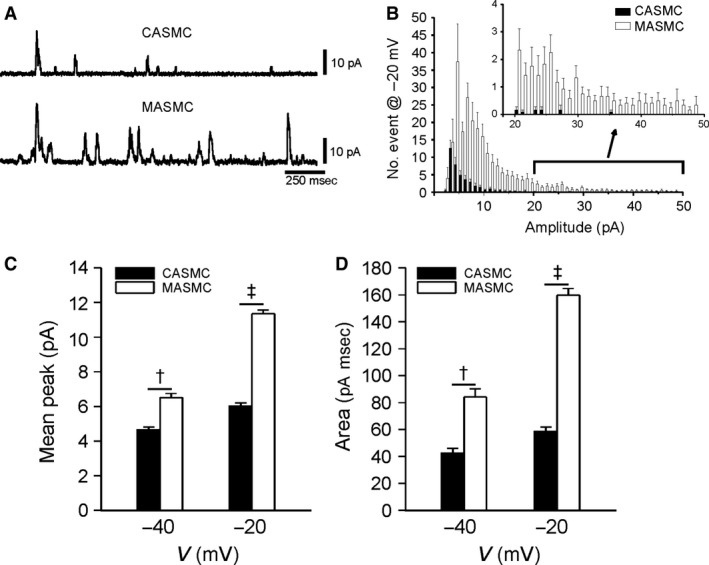
Comparison of STOCs parameters between CASMCs and MASMCs. (A) Representative traces of STOCs detected at −20 mV. STOCs were recorded under whole‐cell configuration with low Ca^2+^ buffer (0.1 mmol/L EGTA) in the pipette solution. (B) Summarized amplitude histogram (1 pA bin size) of STOCs recorded at −20 mV during 1‐min recordings from 12 cells. (C–D) Summary of STOCs mean peak (C) and area (D) obtained from isolated VSMCs at different holding voltages (−40, −20 mV) (^†^ *P* < 0.01, ^‡^ *P* < 0.001).

The STOCs reflecting the BK_Ca_ activity triggered by Ca^2+^ sparks were recorded at −20 mV in the whole‐cell patch clamp with 0.1 mmol/L EGTA in the pipette solution (Fig. 5A). The number of events, mean peak amplitudes, and averaged areas of STOCs were commonly higher in MASMCs than CASMCs (Fig. 5B–D). The mean peak amplitudes of STOCs (Fig. 5C) were calculated by dividing the sum of event amplitudes by the number of STOCs.

**Figure 6:**
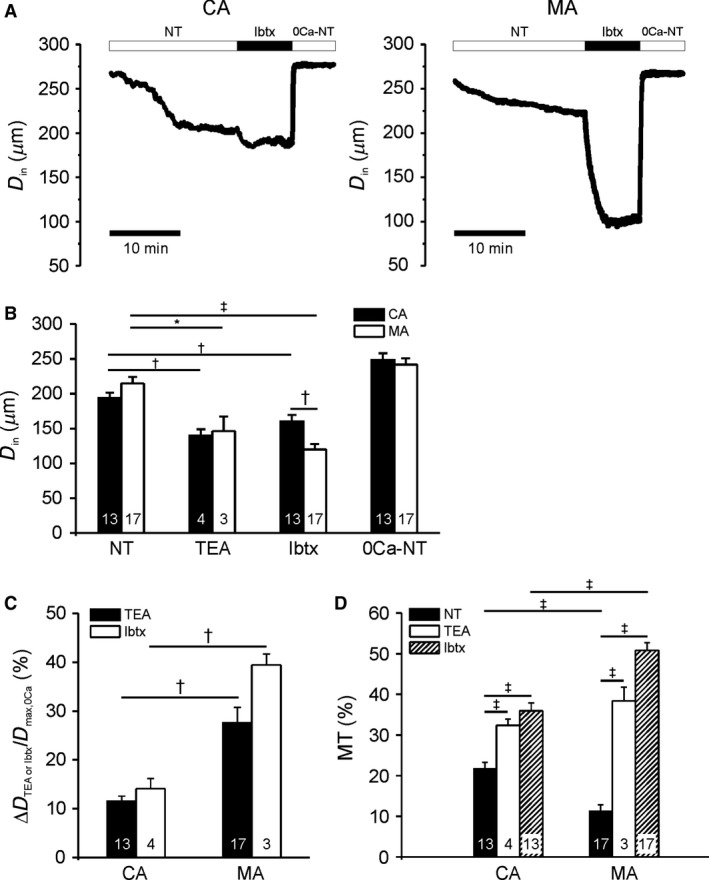
Effects of BK_C__a_ blockers on pressurized CA and MA at 60 mmHg. (A) Representative traces of *D*_in_ change induced by 100 nmol/L iberiotoxin (Ibtx) from CA (left) and MA (right) at 60 mmHg of *P*_lum_. (B) Summary of *D*_in_ in spontaneous MT in control (NT), with 1 mmol/L TEA, with 100 nmol/L Ibtx and 0Ca‐NT conditions (*P < 0.05, ^†^ *P* < 0.01, ‡*P* < 0.001). (C) Summary of normalized *D*_in_ changes (Δ*D*_in_/*D*_max,0Ca_) induced by TEA or Ibtx (^†^ *P* < 0.01). (D) Summary of the MT after the treatment with TEA or Ibtx in comparison with the control MT in NT (^‡^ *P* < 0.001).

Finally, the contribution of BK_Ca_ activity to the MT was examined in MA and CA pressurized to 60 mmHg. Application of iberiotoxin induced more significant constriction in MA than CA (Fig. 6A). We also investigated the effect of additional structurally different maxi‐K channel blocker, TEA. The averaged *D*_in_ levels, the normalized changes in *D*_in_ (Δ*D*_TEA or Ibtx_/D_max,0Ca_), and MT levels with or without 1 mmol/L TEA and 100 nmol/L iberiotoxin were summarized as bar graphs (Fig. 6B–D). The *D*_in_ was decreased by TEA and iberiotoxin treatment in CASMCs and MASMCs, which was more prominent at MASMCs.

## Discussion

This study showed that MT_MA_ is consistently smaller than MT_CA_ despite the higher amplitudes of *I*_CaV_ in MASMCs than in CASMCs. Both *I*_Kv_ and *I*_BKCa_ were higher in MASMCs than in CASMCs. The pharmacological test with iberiotoxin and TEA suggests that the higher BK_Ca_ activity in MASMCs effectively attenuates the MT_MA_, most likely through the hyperpolarizing influence. In contrast to our study, a previous report by Chlopicki et al. showed that the sustained phase of MT_MA_ was weakened by BK_Ca_ inhibitor, charybdotoxin (Chlopicki et al. 2001). However, because the K^+^ channel blocker would induce membrane depolarization, it is very hard to interpret the paradoxical vasorelaxation reported by Chlopicki et al.

Despite the significant density of *I*_Kv_, the application of 4‐AP did not change MT_MA_, indicating relatively small contribution of Kv to the membrane potential of MA under physiological pressure. The *I*_Kv_ in MASMCs might play significant roles under additional depolarizing influences from vasoactive agonists.

The higher density of *I*_BKCa_ in both whole‐cell and inside‐out conditions with fixed [Ca^2+^]_c_ strongly suggests higher level of BK_Ca_ expression in the plasma membrane of MASMCs than CASMCs. Additionally, the Ca^2+^‐sensitivity of BK_Ca_ analyzed in inside‐out configuration also appeared higher in MASMCs than CASMCs (Fig. 3C and D). Because the unitary slope conductance of BK_Ca_ are not different (Fig. 3B), the *α*‐subunit of BK_Ca_ (Slo1*α*) seem to be same between MASMCs and CASMCs (Xia et al. 2002).

According to Yang et al., BK_Ca_ activity in rat CASMCs is higher than skeletal arterial myocytes that was ascribed to the higher ratio of *β*1‐subunit over *α*‐subunit of Slo1 (Yang et al. 2009, 2013). In our hands, the qPCR analysis did not reveal a difference of Slo1*α* and *β*1 subunits between MASMCs and CASMCs (Fig. 3E and F). Unfortunately we could not directly analyze the protein amounts between the small resistance MAs and CAs due to the tiny amounts of myocytes available after the enzymatic single cell isolation. Although we could not conduct immunoblot assays, we performed immunofluorescence confocal microscopy to compare the expression levels of BK_Ca_ subunits. Although the confocal images require careful interpretation in terms of the protein expression levels, the experimental results suggest that Slo1*α* expression appeared higher in MASMCs than CASMCs (Fig. 4A). In both cell types, Slo*β*1 signal was more prominently observed than the Slo1*α*, while no significant difference between MASMCs and CASMCs (Fig. 4B). Taken together, the functional difference of BK_Ca_ current density might be ascribed to the differential expression of Slo1*α*. Also, an unidentified difference in channel modulatory factors might underlie the higher Ca^2+^‐sensitivity of BK_Ca_ in MASMCs, which requires further investigation.

The more prominent STOCs in MASMCs (Fig. 5) might be due to the putatively higher Ca^2+^‐sensitivity of BK_Ca_ and more frequent generation of Ca^2+^ sparks in MASMCs. Because the Ca^2+^ sparks can be triggered by flickering activation of CaV, the higher density of CaV in MASMCs might also contribute to the more frequent STOCs. In contrast to the global [Ca^2+^]_c_ increase, Ca^2+^ sparks negatively regulate contraction of arterial smooth muscle (Jaggar et al. 1998). The physiological role of STOCs became evident from the case of impaired coupling between STOC and Ca^2+^ spark coupling in the arterial myocytes of diabetic rat model (Rueda et al. 2013). A rigorous comparison between STOCs with Ca^2+^ sparks using confocal microscopy combined with patch‐clamp study might provide more clear evidence of the putative differential STOCs/Ca^2+^ spark coupling between different arteries.

### Physiological implication of the different MR and ion channel activities

A previous study of intestinal microcirculation has shown that the microvilli blood flow is effectively autoregulated while less effective in the muscular layer (Davis and Gore 1985). Although the autoregulation of intestinal blood flow is mainly observed at the submucosal arterioles, due to the large volume of intestinal tissue to be perfused, the blood flow through the MA branches would be relatively higher than that of the CA with similar diameter. In this respect, the weak MR_MA_ would be physiologically relevant, and the higher activity of BK_Ca_ would be a compensatory property to keep the low MT_MA_ despite the higher *I*_CaV_. The weak MT_MA_ also implies intrinsically low resistance without vasoactive signals. On the other hand, the low resistance of MA might be more beneficial to effectively redirect the intestinal blood flow for the recruitment under ‘fight and flight’ conditions. Under such conditions, the higher density of *I*_Kv_ might play a role to counterbalance the excessive vasoconstriction of MA (Fig. 2A and B).

In comparison with the other systemic arteries, sympathetic regulation is minimized in the cerebral resistance arteries, and the MT‐dependent autoregulation seems to be the critical mechanism for the constant cerebral blood flow. The marked MT_CA_ coupled with their parallel arrangement would promote regional regulation of flow with minimal overall changes in blood volume. Also, according to LaPlace's law, the myogenic constriction would effectively decrease the wall tension and thus reduces the stimulus for further constriction. Thus, the more sensitive MT_CA_ might enable to maintain a lower wall tension in CA than the similar sizes of MA. Previously, the mechanism underlying such difference may be related to a higher I_CaV_ in CASMCs (Asano et al. 1993). However, our present study showed that the differential activity of K^+^ channels in VSMC might be more critical factor to determine the level of MT.

In summary, our study indicate that MA contain an intrinsic capacity of potent MT that is veiled by higher BK_Ca_ activities. The differential patterns of ion channel currents may provide variability in MT and underlying membrane voltages in the arteries suppling heterogeneous tissues and organs. Physiological and pharmacological modulation of BK_Ca_ would be an effective manner to regulate the intestinal blood flow.

## Conflict of Interest

No conflicts of interest, financial or otherwise, are declared by the authors.
